# Moderating effect of autistic traits on the relationship between peripheral visual processing and facial emotion recognition

**DOI:** 10.3758/s13414-026-03222-x

**Published:** 2026-02-23

**Authors:** Yuki Harada, Nana Kamei, Chiharu Tsukiyama, Kento Shiozaki, Junji Ohyama, Makoto Wada

**Affiliations:** 1 Faculty of Humanities, Kyoto University of Advanced Science, Kyoto city, Kyoto, 615-0096 Japan; 2 Developmental Disorders Section, Department of Rehabilitation for Brain Functions, Research Institute of National Rehabilitation Center for Persons with Disabilities, Tokorozawa, Saitama 359-8555 Japan; 3 Human Augmentation Research Center, National Institute of Advanced Industrial Science and Technology, Kashiwa, Chiba 277-0882 Japan

**Keywords:** Autistic traits, Functional field of view, Facial emotion recognition, Visual processing

## Abstract

Distinct visual processing patterns are one of the underlying mechanisms of atypical facial emotion recognition in individuals with autism spectrum disorder. However, the role of peripheral visual processing, particularly the functional field of view (FFOV), remains unclear. Therefore, this study aimed to examine the relationships among autistic traits, FFOV size, and facial emotion recognition ability. Seventy-five students completed the Autism-Spectrum Quotient (AQ) and then performed facial emotion recognition and FFOV tasks. In the emotion recognition task, participants viewed one of five facial expressions (anger, disgust, fear, happiness, or sadness) on a monitor and selected the word that best described the expression. The FFOV task followed a similar procedure, except that the target digit was presented in the peripheral vision immediately after the facial images disappeared. FFOV size was estimated by fitting psychometric functions to the identification performance of the digits as a function of the target eccentricity. The major findings were: (a) AQ scores did not predict FFOV size, (b) FFOV size was positively correlated with the accuracy of facial emotion recognition, and (c) this correlation became non-significant with lower AQ scores. The findings suggest that peripheral visual processing is associated with facial emotion recognition ability, and that this association varies as a function of autistic traits.

## Introduction

Individuals with autism spectrum disorder (ASD) often exhibit atypical facial emotion recognition (Harada et al., 2024; Law Smith et al., 2010; Wallace et al., 2011), impairing their social communication and interaction. A meta-analysis confirmed the statistical reliability of these atypical emotion recognition patterns, such as recognition difficulties, especially for negative emotional expressions (Uljarevic & Hamilton, 2013). Despite extensive research, the factors contributing to atypical facial emotion recognition in individuals with ASD remain unclear.

One mechanism underlying atypical facial emotion recognition in individuals with autism is their distinct eye-movement patterns. Eye-tracking studies show that autistic individuals spend less time fixating on the eyes (Klin et al., 2002), which are key for conveying social cues (Baron-Cohen et al., 2001a). This reduction in fixation is explained through two accounts. The social salience account attributes it to atypical amygdala responses (Adolphs et al., 2001), resulting in reduced attention to socially salient areas such as the eyes. In contrast, the eye avoidance account suggests that heightened sensitivity to social stimuli, especially angry expressions, causes avoidance to increased arousal (Tanaka & Sung, 2016). Despite different processes, both accounts suggest that reduced eye fixation impairs facial emotion recognition.

Although eye-movement accounts have been used to explain atypical facial emotion recognition, the role of peripheral visual processing, particularly the functional field of view (FFOV), remains unclear. The FFOV refers to the area of the visual field surrounding the fovea in which visual details can be well recognized (Sanders, 1970). FFOV size changes flexibly with cognitive load (Ikeda & Takeuchi, 1975), emotional arousal (Nobata et al., 2010), and aging (Ito et al., 2009). Importantly, individuals with ASD exhibit a narrower FFOV than typically developing individuals (Song et al., 2015). This finding is consistent with the idea that autistic traits enhance detailed processing (Plaisted et al., 1999) and local information (Bölte et al., 2007).

The FFOV determines the amount of information that can be simultaneously processed in the peripheral visual field, suggesting that it constrains the degree to which multiple facial features can be integrated into a holistic perception. Holistic processing – essential in facial recognition (Farah et al., 1995) – can occur in peripheral vision (Cañas-Bajo & Whitney, 2022; Kovács et al., 2017), although its strength is generally weaker than in central vision. As FFOV shrinkage limits access to peripheral facial features such as the eyes, eyebrows, and mouth, it likely promotes piecemeal rather than holistic processing. Indeed, holistic processing is reduced when some parts of the face are obscured (Leong et al., 2023). Despite its theoretical relevance, however, the relationship between FFOV size and facial emotion recognition ability has yet to be empirically examined.

Moreover, the effect of FFOV size on facial emotion recognition may be mediated by autistic traits. Nakano et al. (2010) found that individuals with ASD inaccurately recognized objects when only a part of the object was presented through a narrow slit, but correctly recognized them when the entire image was shown. This finding is consistent with the theory of weak central coherence, which suggests that autistic individuals have difficulty integrating partial details into a coherent whole (Happé & Frith, 2006). When FFOV is restricted, individuals focus on isolated facial features, such as the eyes, nose, and mouth, rather than the face. In such cases, FFOV shrinkage might impair facial recognition, particularly in autistic individuals.

This study investigated the relationships among autistic traits, FFOV size, and facial emotion recognition ability. Non-diagnosed students completed the Autism Spectrum Quotient (AQ) and performed two tasks evaluating facial emotion recognition ability and FFOV size. We hypothesize that (Hypothesis 1) autistic traits would shrink the FFOV, (Hypothesis 2) FFOV size would be positively correlated with the ability to recognize facial emotions, and (Hypothesis 3) autistic traits would moderate the relationship between FFOV size and facial emotion recognition accuracy.

## Methods

### Participants

Seventy-five university students (34 males and 41 females) from Japan participated in this experiment. Their ages ranged from 18 to 36 years (M = 20.747 years, SD = 2.269 years). A priori power analysis was conducted using G*Power 3.0 with the following parameters: test family = *t*-tests, statistical test = linear multiple regression: fixed model, single regression coefficient, two-tailed test, Effect size f^2^ = .10, α = .05, power (1 − β) = .80, and three predictors. Although our sample size was slightly below the required size, it would retain sufficient sensitivity to detect small-to-moderate effects in the mixed-model analyses. All participants had normal or corrected-to-normal visual acuity. Written informed consent was obtained from all participants. The local ethics committee of the first author’s institution approved the study.

### Materials and apparatus

Participants’ autistic traits were assessed using the Japanese version of the AQ (Wakabayashi et al., 2004), translated from the original scale (Baron-Cohen et al., 2001b). The questionnaire consists of 50 items covering five domains: social skills, attentional shifts, local processing, communication, and imagination. Each item was rated on a four-point scale (agree, partially agree, partially disagree, and disagree). Autistic traits were evaluated by scoring the responses “agree” and “partially agree” to autistic items and the responses “disagree” and “partially disagree” to inverted autistic items.

Facial images depicting anger, disgust, fear, happiness, and sadness were selected from the Facial Database of Advanced Industrial Science and Technology (Fujimura & Umemura, 2018). The emotion of surprise was excluded because it was considered neutral in terms of valence and reduced the number of experimental trials, thereby minimizing the effect of emotional adaptation. A total of 30 facial images were used in this experiment: five emotions × six individuals (three males and three females).

The apparatus included a laptop PC, 24-in. LCD monitor, and eye tracker (Tobii Pro Nano, Tobii). The eye tracker was used to measure the size of the FFOV by using a gaze-contingent procedure. The experiment was programmed in MATLAB (MathWorks, Natick, MA, USA) using PsychToolbox (Kleiner et al., 2007).

### Procedure

The participants were instructed with regard to the experimental procedure and provided written informed consent. After completing the AQ, they performed the facial emotion recognition and FFOV tasks. Their heads were stabilized using a chin rest to maintain a viewing distance of 57 cm.

**Fig. 1:**
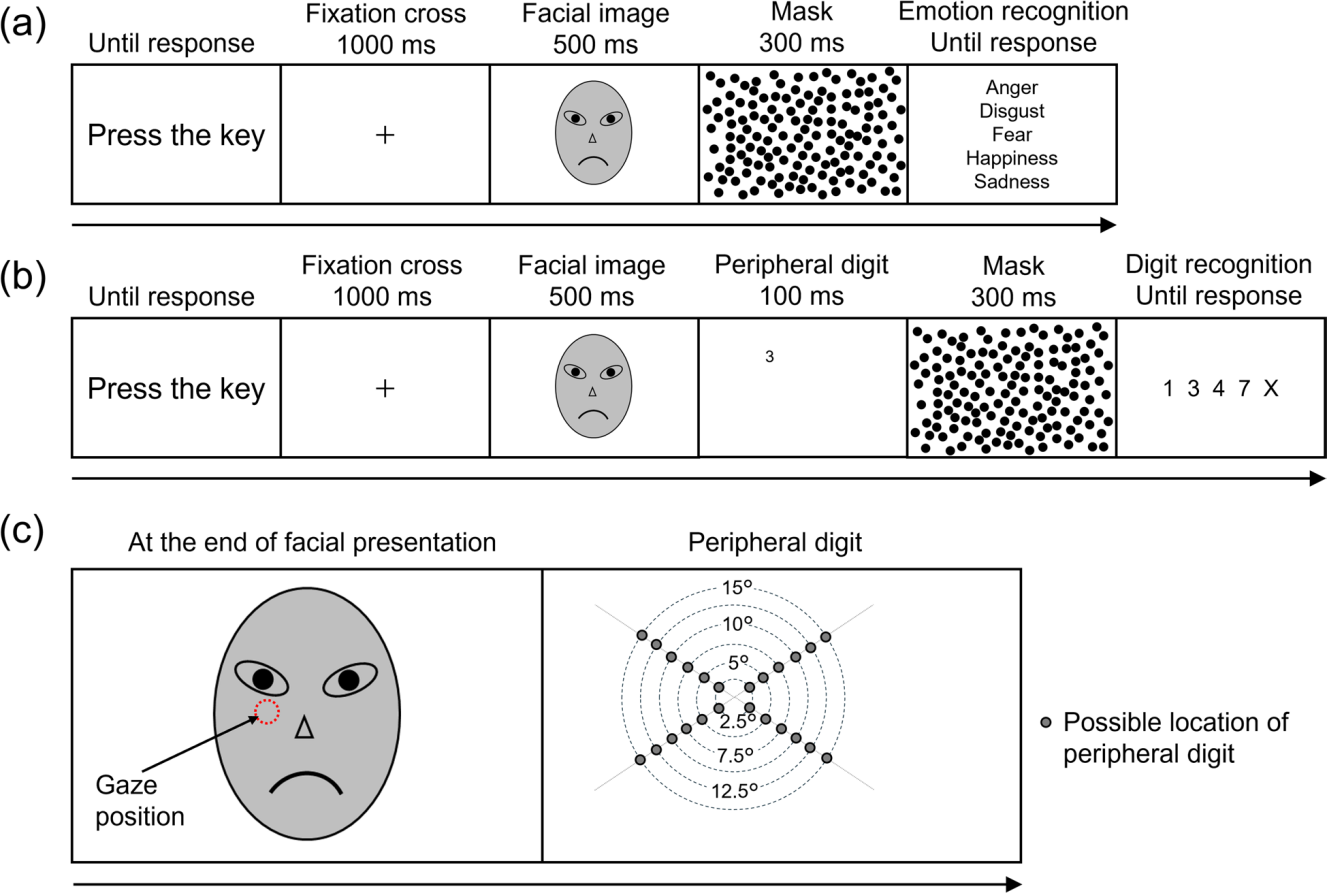
Schematic illustration of trial sequence in experimental tasks. (**a**) The trial sequence of the facial emotion recognition task. (**b**) The trial sequence of the functional field of view (FFOV) task. (**c**) The determination of digit location. A peripheral digit was presented along a line extending in one of four diagonal directions from the recorded gaze position. Real facial images were replaced with schematic illustrations owing to restrictions on photograph publication imposed by the license agreement

In the facial emotion recognition task, the participants were asked to select the word that best described the presented facial expressions. The trial sequence is shown in Fig. 1a. Each trial began when the participants pressed the space key, presenting a fixation cross (“+”) for 1,000 ms. Subsequently, a facial image (25° of visual angle in width, 30° in height) was presented for 500 ms. Then, the random dot mask covered the entire screen. The participants then selected the emotion word that best described the presented facial image from five forced-choice alternatives: anger, disgust, fear, happiness, or sadness. No rapid response was required. The next trial began at the end of the trial period. The order of the facial emotions was randomized across participants. This task consisted of 60 trials: five facial emotions × six individuals × two repetitions.

In the FFOV task, participants were asked to identify a digit presented in their peripheral vision immediately following the offset of the facial images. Before beginning the task, participants completed a five-point calibration using an eye tracker. FFOV size was assessed using a gaze-contingent procedure that dynamically controlled and manipulated the location of peripheral digits based on participants’ real-time gaze positions. Notably, this procedure manipulated only the eccentricity of peripheral digits, not the facial images, ensuring consistent retinal eccentricity for each trial. This control was essential for obtaining an accurate and precise measurement of FFOV size.

The trial sequence for the FFOV task is shown in Fig. 1b. The sequence was similar to that of the facial emotion recognition task, except for the presentation and response to the peripheral digits. At the end of the facial presentation, participants’ gaze position was recorded using the eye tracker. Then, a peripheral digit appeared along one of four diagonal lines extending from the recorded gaze position (Fig. 1c). The retinal eccentricity of the peripheral digit was manipulated across six levels (2.5°, 5°, 7.5°, 10°, 12.5°, and 15° visual angles). After the random dot mask, the participants selected from five alternatives (1, 3, 4, 7, and “I could not recognize it”). Because FFOV shrinkage makes peripheral digit recognition more difficult, performance on this task would evaluate the size of the FFOV. The order of facial emotions and digit eccentricity was randomized across participants. The total number of trials was 180: five facial emotions × six individuals × six digit eccentricities.

Facial images were presented in the FFOV task because emotionally salient stimuli can modulate FFOV size (Harada et al., 2020; Nobata et al., 2010). As the present study aimed to examine whether FFOV size is associated with facial emotion recognition, it was important to measure FFOV within a comparable facial context. Although both tasks involved facial stimuli, their cognitive demands differed: the FFOV task required peripheral digit identification without explicit emotion processing, whereas the facial emotion recognition task required explicit emotion judgments. Using similar visual content thus enhanced ecological validity while maintaining distinct task demands.

### Analysis

The independent variables were the AQ score, facial emotion (anger, disgust, fear, happiness, and sadness), and digit eccentricity (2.5°, 5°, 7.5°, 10°, 12.5°, and 15° visual angles, applicable only to the FFOV task). The dependent variables were the accuracy of facial emotion recognition and digit identification.

**Fig. 2:**
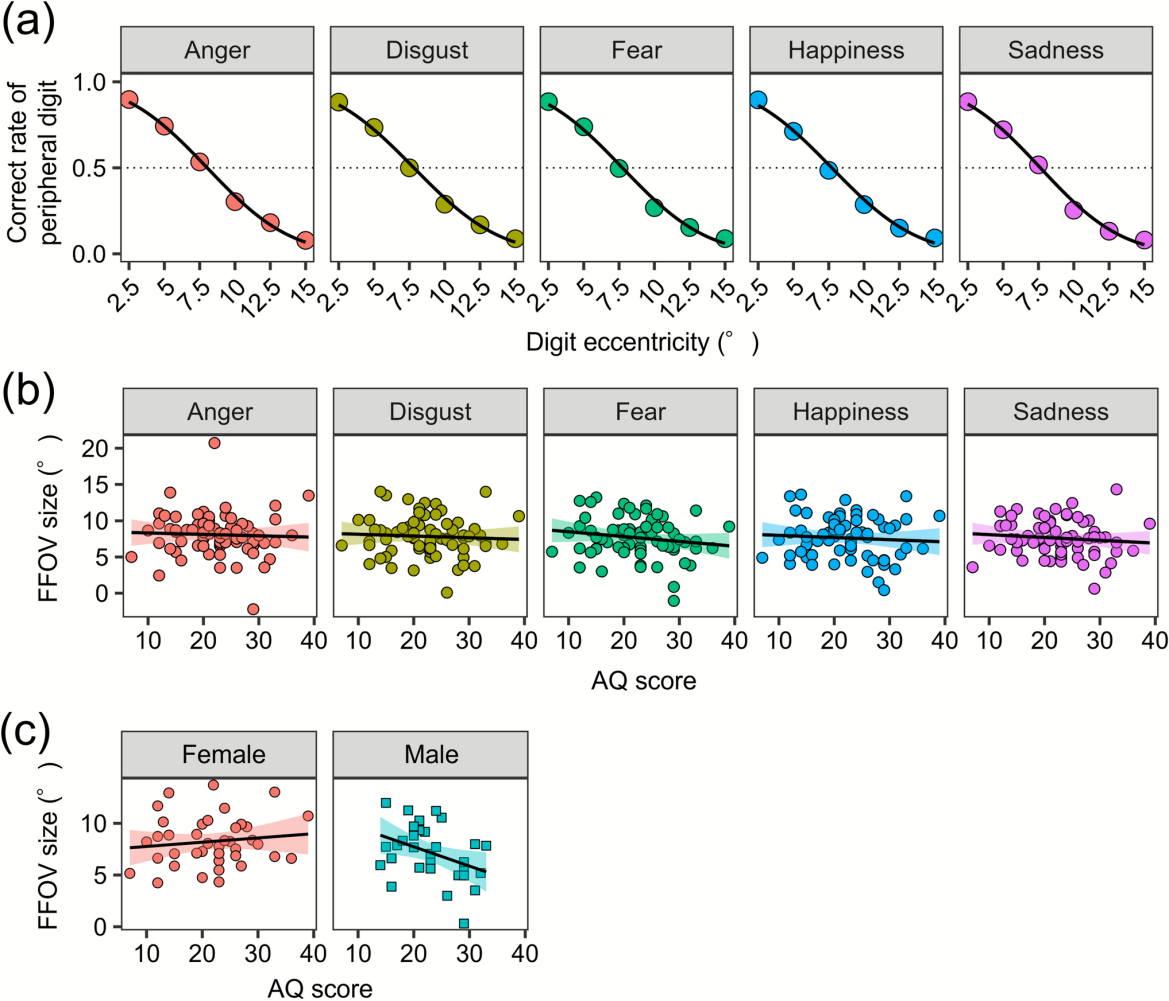
Data from the functional field of view (FFOV) task. (**a**) The mean correct rate of digit identification, calculated from data pooled across participants. The curve lines represent fitted cumulative Gaussian functions. The horizontal dotted lines indicate the thresholds at which the correct rate was .5, while the vertical dotted lines indicate FFOV size. (**b**) The effect of the Autism-Spectrum Quotient (AQ) score on FFOV size across facial emotions. (**c**) The effect of AQ score on FFOV size across participants’ sex

FFOV size was evaluated for each facial emotion and each participant, as it can vary depending on the emotional content within the same individual (Harada et al., 2020). For each participant and facial emotion, a cumulative Gaussian function was fitted to the digit-identification data as a function of digit eccentricity, using the maximum-likelihood method. Figure 2a shows the mean digit identification accuracy and fitted functions that were calculated by pooled data across participants. FFOV size was operationally defined as the threshold at which the correct identification rate reached .5, following the approaches of Song et al. (2015).

Before analysis, trials without peripheral digit presentation owing to undetected gaze at facial offset were excluded. Data from six participants were excluded as statistical outliers of FFOV size (*M* ± 3 *SD*) in any emotion condition. Such outliers are frequently caused by inaccurate eye tracking. Continuous variables (FFOV size and AQ score) were centered before analysis. Linear mixed-model analyses were conducted using Jeffreys’ Amazing Statistics Program (version 0.19.3: JASP Team, 2025) to test the three hypotheses.

## Results

### Effect of autistic traits on the size of the FFOV

Figure 2b shows the mean FFOV size across facial emotion conditions. To examine the effect of autistic traits on FFOV size, a linear mixed-model analysis was performed, including AQ scores, facial emotions, and their interaction as fixed effects, with participants as a random effect. The results showed that neither the main effects nor their interaction were significant (*F*s < 0.657, *p*s >.437). Autistic traits did not reduce FFOV, which is inconsistent with Hypothesis 1.

Previous studies have reported varied attentional characteristics between male and female individuals with ASD (Bölte et al., 2011). To examine the effect of autistic traits on FFOV size for each sex, we performed a post hoc linear mixed-model analysis of FFOV size, including facial emotion, AQ score, and sex (male, female) as fixed effects, with participants as a random effect. The results indicate a significant interaction between AQ scores and sex [*F* (1, 66.00) = 5.645, *p* =.020], while the other main effects and interactions were not significant (*F*s < 3.351, *p*s >.072). A simple slope analysis (Fig. 2c) revealed that FFOV size was negatively correlated with the AQ scores in male participants (*β* = −0.187, *SE* = 0.080, *z* = −2.333, *p* =.020) but not in female participants (*β* = 0.041, *SE* = 0.053, *z* = 0.779, *p* =.436).

### Associations among autistic traits, FFOV size, and facial emotion recognition

A linear mixed-model analysis on recognition accuracy was conducted. The AQ score, facial emotion, FFOV size, and their two- and three-way interactions were included as fixed effects, with participants as a random effect. The results revealed significant main effects of FFOV size [*F* (1, 111.33) = 5.216, *p* =.024] and facial emotion [*F* (4, 266.56) = 60.477, *p* <.001], whereas the effect of the AQ score was not significant [*F* (1, 67.28) = 0.009, *p* =.926]. This supports the notion that FFOV size is positively correlated with facial emotion recognition accuracy (Hypothesis 2). Additionally, significant interactions were found between the AQ score and FFOV size [*F* (1, 118.17) = 4.577, *p* =.034] and between FFOV and facial emotion [*F* (4, 266.42) = 2.822, *p* =.025]. The other interactions were not significant (*F*s < 1.912, *p*s >.109).

**Fig. 3:**
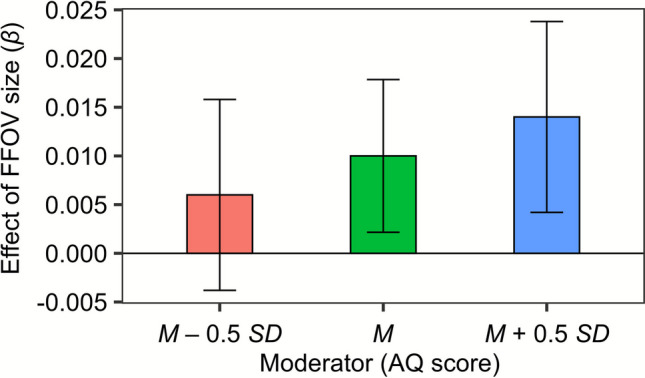
The simple slopes of functional field of view (FFOV) size predicting emotion recognition accuracy at three Autism-Spectrum Quotient (AQ) levels (*M* – 0.5 *SD*, *M*, and *M* + 0.5 *SD*). Note that the AQ score was treated as a continuous variable in the analysis. Error bars represent 95% confidence intervals

For the interaction between the AQ score and FFOV size, a simple slope analysis revealed that facial emotion recognition accuracy declined more significantly with decreasing FFOV size at AQ = *M* (*β* = 0.010, *SE* = 0.004, *z* = 2.284, *p* =.022) and AQ = *M* + 0.5 *SD* (*β* = 0.014, *SE* = 0.005, *z* = 3.080, *p* =.002), whereas no significant decline was observed at AQ = *M* – 0.5 *SD* (*β* = 0.006, *SE* = 0.005, *z* = 1.112, *p* =.266) (Fig. 3). These results support Hypothesis 3. Furthermore, for the interaction between FFOV and facial expression, we performed a simple slope analysis. The results indicated that recognition accuracy declined more significantly with decreasing FFOV size for disgust (*β* = 0.033, *SE* = 0.009, *z* = 3.731, *p* <.001) but not for the other four expressions (*β*s < 0.013, *SE*s > 0.008, |*z|*s < 1.340, *p*s >.180).

## Discussion

The present study examined the relationships between autistic traits, FFOV size, and facial emotion recognition. The results indicated (a) no effect of autistic traits on the facial emotion recognition accuracy, (b) a significant positive correlation between FFOV size and facial emotion recognition accuracy, and (c) a significant interaction between FFOV size and AQ scores on facial emotion recognition accuracy. These results support Hypotheses 2 and 3, but not Hypothesis 1.

One major finding is the significant interaction between FFOV size and autistic traits on facial emotion recognition accuracy. Specifically, recognition accuracy was more positively correlated with FFOV size as participants’ autistic traits increased (Fig. 3). This pattern can be explained by the theory of weak central coherence in autistic individuals. When FFOV is restricted, individuals perceive limited facial details at each fixation and temporally integrate them to recognize facial emotions. However, autistic individuals exhibit a weak central coherence (Happé & Frith, 2006; Smith et al., 2015), which impairs their ability to integrate local visual details into global information. Consequently, they may struggle to integrate an entire expression from restricted facial details. This interpretation aligns with previous findings indicating that individuals with ASD had greater difficulty recognizing familiar objects when presented in fragmented form, whereas no such difficulty was observed when full images were shown (Nakano et al., 2010).

Our findings broaden the understanding of visual processing in atypical facial emotion recognition among autistic individuals. While previous studies have primarily focused on gaze patterns, such as reduced fixations on eyes (Tanaka & Sung, 2016), our findings underscore the additional role of peripheral visual processing. The coordination between gaze patterns and peripheral visual processing may be key to understanding facial emotion recognition in autistic individuals. During scene viewing, eye movements are influenced by peripheral processing. For instance, individuals with narrower FFOV compensate with more frequent saccadic movements (Beurskens & Bock, 2011). However, autistic individuals may show reduced compensatory behavior due to autistic inertia, which refers to a tendency to experience difficulties initiating and shifting actions (Buckle et al., 2021). Given this inertia, gaze-peripheral coordination may differ between autistic and typically developing individuals.

The results also showed that autistic traits are associated with the FFOV size in males. This finding is partially consistent with Hypothesis 1 and Song et al. (2015). Given that most participants in Song et al. were male (12 males and one female), their findings likely reflect the characteristics of males. Autistic males focus strongly on detailed visual information (Bölte et al., 2011). Contrastingly, autistic traits did not impact FFOV size in female participants. Previous studies have suggested that attentional characteristics in females with ASD are relatively typical compared to those in males with ASD. For example, performance in the Embedded Figure Test did not differ between females with and without ASD, whereas it was worse in males with ASD (Lai et al., 2012). Although our analyses are exploratory and conducted post hoc, these findings suggest potential sex differences that warrant further investigation.

The present study has two notable limitations. First, we employed correlational analyses to examine the relationships among autistic traits, FFOV size, and facial emotion recognition ability. However, these analyses do not allow for causal inferences and cannot rule out the influence of other cognitive factors, such as the theory of mind and executive function. Previous studies have suggested that individuals with ASD show impairments in theory of mind (Baron-Cohen et al., 1985) and executive function (Ozonoff et al., 1991). Furthermore, theory of mind has been linked to facial emotion recognition (Lee et al., 2014), while executive function has been associated with FFOV size (Ball et al., 2007). These findings suggest that unmeasured cognitive factors may mediate the observed associations, limiting the ability to draw definitive conclusions about causality. Second, we recruited non-diagnosed individuals and AQ scores to assess autistic traits. While autistic traits exist continuously (Abu-Akel et al., 2019), AQ scores might not accurately capture these traits in typically developing individuals (Lundqvist & Lindner, 2017). Additionally, the social characteristics of individuals with clinical diagnoses may differ from those with only self-reported traits. Therefore, future research should further investigate the relationship between autistic traits, FFOV size, and facial emotion recognition using more refined visual techniques (e.g., gaze-contingent window procedures) and clinically diagnosed ASD samples.

Moreover, the present results do not provide evidence about the relationship between autistic traits and the spatial asymmetry of the FFOV. Autistic individuals tend to fixate more on the mouth than the eyes in facial processing (Klin et al., 2002), suggesting that their FFOV may be biased toward the mouth rather than the eyes. This mouth-biased FFOV could interact with facial expression type, given known differences in attentional allocation depending on emotional faces (Eisenbarth & Alpers, 2011). However, we were unable to explore this possibility, as the direction of peripheral digit presentation relative to the fovea was not manipulated. To test potential asymmetries in FFOV among autistic individuals, future studies should systematically manipulate the direction of peripheral targets (e.g., upper, lower, left, and right visual fields). Such a procedure would provide a more comprehensive understanding of how peripheral visual processing supports social and emotional cognition.

## Data Availability

The datasets and codes of this study can be found on the Open Science Framework at: https://osf.io/jeurk/files/osfstorage.

## References

[CR1] Abu-Akel, A., Allison, C., Baron-Cohen, S., & Heinke, D. (2019). The distribution of autistic traits across the autism spectrum: Evidence for discontinuous dimensional subpopulations underlying the autism continuum. *Molecular Autism,**10*, Article 24. 10.1186/s13229-019-0275-3 31149329 10.1186/s13229-019-0275-3 PMC6537408

[CR2] Adolphs, R., Sears, L., & Piven, J. (2001). Abnormal processing of social information from faces in autism. *Journal of Cognitive Neuroscience,**13*, 232–240. 10.1162/089892901564289 11244548 10.1162/089892901564289

[CR3] Ball, K., Edwards, J. D., & Ross, L. A. (2007). The impact of speed of processing training on cognitive and everyday functions. *The Journal of Gerontology. Series B, Psychological Sciences and Social Sciences,**62B*, 19–31. 10.1093/geronb/62.special_issue_1.19 10.1093/geronb/62.special_issue_1.19 17565162

[CR4] Baron-Cohen, S., Leslie, A. M., & Frith, T. (1985). Does the autistic child have a “theory of mind”? *Cognition,**21*, 37–46. 10.1016/0010-0277(85)90022-8 2934210 10.1016/0010-0277(85)90022-8

[CR5] Baron-Cohen, S., Wheelwright, S., Hill, J., Raste, Y., & Plumb, I. (2001). The “Reading the Mind in the Eyes” test revised version: A study with normal adults, and adults with Asperger syndrome or high-functioning autism. *Journal of Child Psychology and Psychiatry, and Allied Disciplines,**42*, 241–251. 10.1111/1469-7610.00715 11280420

[CR6] Baron-Cohen, S., Wheelwright, S., Skinner, R., Martin, J., & Clubley, E. (2001). The Autism-Spectrum Quotient (AQ): Evidence from Asperger syndrome/high-functioning autism, males and females, scientists and mathematicians. *Journal of Autism and Developmental Disorders,**31*, 5–17. 10.1023/a:1005653411471 11439754 10.1023/a:1005653411471

[CR7] Beurskens, R., & Bock, O. (2011). Age-related decline of peripheral visual processing: The role of eye movements. *Experimental Brain Research,**217*, 117–124. 10.1007/s00221-011-2978-3 22179529 10.1007/s00221-011-2978-3 PMC3279647

[CR8] Bölte, S., Duketis, E., Poustka, F., & Holtmann, M. (2011). Sex differences in cognitive domains and their clinical correlates in higher-functioning autism spectrum disorders. *Autism,**15*, 497–511. 10.1177/1362361310391116 21454389 10.1177/1362361310391116

[CR9] Bölte, S., Holtmann, M., Poustka, F., Scheurich, A., & Schmidt, L. (2007). Gestalt perception and local-global processing in high-functioning autism. *Journal of Autism and Developmental Disorders,**37*, 1493–1504. 10.1007/s10803-006-0231-x 17029017 10.1007/s10803-006-0231-x

[CR10] Buckle, K. L., Leadbitter, K., Poliakoff, E., & Gowen, E. (2021). No way out except from external intervention”: First-hand accounts of autistic inertia. *Frontiers in Psychology,**12*, Article 631596. 10.3389/fpsyg.2021.631596 34326790 10.3389/fpsyg.2021.631596 PMC8314008

[CR11] Cañas-Bajo, T., & Whitney, D. (2022). Relative tuning of holistic face processing towards the fovea. *Vision Research,**197*, Article 108049. 10.1016/j.visres.2022.108049 35461170 10.1016/j.visres.2022.108049 PMC10101769

[CR12] Eisenbarth, H., & Alpers, G. W. (2011). Happy mouth and sad eyes: Scanning emotional facial expressions. *Emotion,**11*, 860–865. 10.1037/a0022758 21859204 10.1037/a0022758

[CR13] Farah, M. J., Tanaka, J. W., & Drain, H. M. (1995). What causes the face inversion effect? *Journal of Experimental Psychology. Human Perception and Performance,**21*, 628–634. 10.1037/0096-1523.21.3.628 7790837 10.1037//0096-1523.21.3.628

[CR14] Fujimura, T., & Umemura, H. (2018). Development and validation of a facial expression database based on the dimensional and categorical model of emotions. *Cognition and Emotion,**32*, 1663–1670. 10.1080/02699931.2017.1419936 29334821 10.1080/02699931.2017.1419936

[CR15] Happé, F., & Frith, U. (2006). The weak coherence account: Detail-focused cognitive style in autism spectrum disorders. *Journal of Autism and Developmental Disorders,**36*, 5–25. 10.1007/s10803-005-0039-0 16450045 10.1007/s10803-005-0039-0

[CR16] Harada, Y., Mitsudo, H., & Ohyama, J. (2020). The effect of unusualness on the functional field of view in unsafe scenes. *Visual Cognition,**28*, 73–85. 10.1080/13506285.2020.1718817

[CR17] Harada, Y., Ohyama, J., Sano, M., Ishii, N., Maida, K., Wada, M., & Wada, M. (2024). Temporal characteristics of facial ensemble in individuals with autism spectrum disorder: Examination from arousal and attentional allocation. *Frontiers in Psychiatry,**15*, Article 1328708. 10.3389/fpsyt.2024.1328708 38439795 10.3389/fpsyt.2024.1328708 PMC10910007

[CR18] Ikeda, M., & Takeuchi, T. (1975). Influence of foveal load on the functional visual field. *Perception & Psychophysics,**18*, 255–260. 10.3758/BF03199371

[CR19] Ito, N., Sagawa, K., & Fukunaga, Y. (2009). Useful visual field at a homogeneous background for old and young subjects. *Gerontechnology,**8*, 42–51. 10.4017/gt.2009.08.01.010.00

[CR20] JASP Team (2025). JASP (version 0.19.3) [Computer software]. https://jasp-stats.org/

[CR21] Kleiner, M., Brainard, D., Pelli, D., Ingling, A., Murray, R., & Broussard, C. (2007). What’s new in psychtoolbox-3? *Perception,**36*, 1–16. 10.1068/v070821

[CR22] Klin, A., Jones, W., Schultz, R., Volkmar, F., & Cohen, D. (2002). Visual fixation patterns during viewing of naturalistic social situations as predictors of social competence in individuals with autism. *Archives of General Psychiatry,**59*, 809–816. 10.1001/archpsyc.59.9.809 12215080 10.1001/archpsyc.59.9.809

[CR23] Kovács, P., Knakker, B., Hermann, P., Kovács, G., & Vidnyánszky, Z. (2017). Face inversion reveals holistic processing of peripheral faces. *Cortex,**97*, 81–95. 10.1016/j.cortex.2017.09.020 29096198 10.1016/j.cortex.2017.09.020

[CR24] Lai, M., Lombardo, M. V., Ruigrok, A. N. V., Chakrabarti, B., Wheelwright, S. J., Auyeung, B., Allison, C., Consortium, M. A., & Baron-Cohen, S. (2012). Cognition in males and females with autism: Similarities and differences. *PLoS One,**7*, Article e47198. 10.1371/journal.pone.0047198 23094036 10.1371/journal.pone.0047198 PMC3474800

[CR25] Law Smith, M. J., Montagne, B., Perrett, D. I., Gill, M., & Gallagher, L. (2010). Detecting subtle facial emotion recognition deficits in high-functioning autism using dynamic stimuli of varying intensities. *Neuropsychologia,**48*, 2777–2781. 10.1016/j.neuropsychologia.2010.03.008 20227430 10.1016/j.neuropsychologia.2010.03.008

[CR26] Lee, S. B., Koo, S. J., Song, Y. Y., Lee, M. K., Jeong, Y., Kwon, C., Park, K. R., Park, J. Y., Kang, J. I., Lee, E., & An, S. K. (2014). Theory of mind as a mediator of reasoning and facial emotion recognition: Findings from 200 healthy people. *Psychiatry Investigation,**11*, 105–111. 10.4306/pi.2014.11.2.105 24843363 10.4306/pi.2014.11.2.105 PMC4023082

[CR27] Leong, B. Q. Z., Estudillo, A. J., & Ismail, A. M. H. (2023). Holistic and featural processing’s link to face recognition varies by individual and task. *Scientific Reports,**13*, Article 16869. 10.1038/s41598-023-44164-w 37803085 10.1038/s41598-023-44164-w PMC10558561

[CR28] Lundqvist, L. O., & Lindner, H. (2017). Is the autism-spectrum quotient a valid measure of traits associated with the autism spectrum? A Rasch validation in adults with and without autism spectrum disorders. *Journal of Autism and Developmental Disorders,**47*, 2080–2091. 10.1007/s10803-017-3128-y 28425021 10.1007/s10803-017-3128-y PMC5487751

[CR29] Nakano, T., Ota, H., Kato, N., & Kitazawa, S. (2010). Deficit in visual temporal integration in autism spectrum disorders. *Proceedings of the Royal Society B: Biological Sciences,**277*, 1027–1030. 10.1098/rspb.2009.1713 10.1098/rspb.2009.1713 PMC2842756 19955150

[CR30] Nobata, T., Hakoda, Y., & Ninose, Y. (2010). The functional field of view becomes narrower while viewing negative emotional stimuli. *Cognition and Emotion,**24*, 886–891. 10.1080/02699930902955954

[CR31] Ozonoff, S., Pennington, B. F., & Rogers, S. J. (1991). Executive function deficits in high-functioning autistic individuals: Relationship to theory of mind. *Journal of Child Psychology and Psychiatry,**32*, 1081–1105. 10.1111/j.1469-7610.1991.tb00351.x 1787138 10.1111/j.1469-7610.1991.tb00351.x

[CR32] Plaisted, K., Swettenham, J., & Rees, L. (1999). Children with autism show local precedence in a divided attention task and global precedence in a selective attention task. *Journal of Child Psychology and Psychiatry, and Allied Disciplines,**40*, 733–742. 10.1111/1469-7610.00489 10433407

[CR33] Sanders, A. F. (1970). Some aspects of the selective process in the functional visual field. *Ergonomics,**13*, 101–117. 10.1080/00140137008931124 5416864 10.1080/00140137008931124

[CR34] Smith, D., Ropar, D., & Allen, H. A. (2015). Visual integration in autism. *Frontiers in Human Neuroscience,**9*, Article 387. 10.3389/fnhum.2015.00387 26190994 10.3389/fnhum.2015.00387 PMC4486830

[CR35] Song, Y., Hakoda, Y., Sanefuji, W., & Cheng, C. (2015). Can they see it? The functional field of view is narrower in individuals with autism spectrum disorders. *PLoS One,**10*, Article e0133237. 10.1371/journal.pone.0133237 26204121 10.1371/journal.pone.0133237 PMC4512679

[CR36] Tanaka, J. W., & Sung, A. (2016). The “eye avoidance” hypothesis of autism face processing. *Journal of Autism and Developmental Disorders,**46*, 1538–1552. 10.1007/s10803-013-1976-7 24150885 10.1007/s10803-013-1976-7 PMC3997654

[CR37] Uljarevic, M., & Hamilton, A. (2013). Recognition of emotions in autism: A formal meta-analysis. *Journal of Autism and Developmental Disorders,**43*, 1517–1526. 10.1007/s10803-012-1695-5 23114566 10.1007/s10803-012-1695-5

[CR38] Wakabayashi, A., Tojo, Y., Baron-Cohen, S., & Wheelwright, S. (2004). The autism-spectrum quotient (AQ) Japanese version: Evidence from high-functioning clinical group and normal adults. *Shinrigaku Kenkyu (Japanese version),**75*, 78–84. 10.4992/jjpsy.75.78 10.4992/jjpsy.75.78 15724518

[CR39] Wallace, G. L., Case, L. K., Harms, M. B., Silvers, J. A., Kenworthy, L., & Martin, A. (2011). Diminished sensitivity to sad facial expressions in high functioning autism spectrum disorders is associated with symptomatology and adaptive functioning. *Journal of Autism and Developmental Disorders,**41*, 1475–1486. 10.1007/s10803-010-1170-0 21347615 10.1007/s10803-010-1170-0 PMC3448486

